# Moving forward with the loss of a loved one: treating PTSD following traumatic bereavement with cognitive therapy

**DOI:** 10.1017/S1754470X23000041

**Published:** 2023-04-20

**Authors:** Jennifer Wild, Michael Duffy, Anke Ehlers

**Affiliations:** 1 Department of Experimental Psychology, University of Oxford, Oxford, UK; 2 Oxford Health NHS Foundation Trust, Oxford, UK; 3 Phoenix Australia, Department of Psychiatry, University of Melbourne, 161 Barry Street, Melbourne, Victoria 3053, Australia; 4 Queen’s University Belfast, UK

**Keywords:** cognitive therapy, grief, imagery, loss trauma, PTSD, traumatic bereavement

## Abstract

Traumatic loss is associated with high rates of post-traumatic stress disorder (PTSD) and appears to inhibit the natural process of grieving, meaning that patients who develop PTSD after loss trauma are also at risk of experiencing enduring grief. Here we present how to treat PTSD arising from traumatic bereavement with cognitive therapy (CT-PTSD; Ehlers *et al*., 2005). The paper describes the core components of CT-PTSD for bereavement trauma with illustrative examples, and clarifies how the therapy differs from treating PTSD associated with trauma where there is no loss of a significant other. A core aim of the treatment is to help the patient to shift their focus from loss to what has not been lost, from a focus on their loved one being gone to considering how they may take their loved one forward in an abstract, meaningful way to achieve a sense of continuity in the present with what has been lost in the past. This is often achieved with imagery transformation, a significant component of the memory updating procedure in CT-PTSD for bereavement trauma. We also consider how to approach complexities, such as suicide trauma, loss of a loved one in a conflicted relationship, pregnancy loss and loss of life caused by the patient.

**Key learning aims:**

1. To be able to apply Ehlers and Clark’s (2000) cognitive model to PTSD arising from bereavement trauma.
2. To recognise how the core treatment components differ for PTSD associated with traumatic bereavement than for PTSD linked to trauma where there is no loss of life.
3. To discover how to conduct imagery transformation for the memory updating procedure in CT-PTSD for loss trauma.

‘*The image I carry forward captures his warmth and his love for life. It makes me remember him from before the trauma. It makes me smile. What more could you ask? You remember your loved one and you smile. With what’s happened, that’s a really good result*.’

Female, 51 years old

## Introduction

Every year approximately 500,000 people die in the UK, leaving behind on average five close loved ones (Zhou *et al*., 2005). Sudden traumatic losses, such as death by suicide or violence or death by protracted illness, increase risk for post-traumatic stress (PTSD) and prolonged grief disorders (PGD). Whilst PTSD in such circumstances is characterised by persistent re-experiencing of the loss trauma, prolonged grief disorder is characterised by the experience of yearning for the deceased. The disorders can co-occur and research suggests that PTSD, in the absence of treatment, interrupts the natural course of grief, increasing risk for PGD (Glad *et al*., 2022). For both disorders, the patient is likely to experience distressing images of the person who died, which may relate to memories of the moments of death, painful points during an illness, including moments indicating a change for the worse (Smith *et al*., 2020), or suffering the patient perceives the person to have experienced. In PGD, the images may also reflect positive memories which now elicit feelings of loss (Smith *et al*., 2020). Cognitive therapy for PTSD (CT-PTSD; Ehlers *et al*., 2005) is a highly effective treatment for PTSD arising from a range of traumas including traumatic loss and is recommended as a first-line treatment for the disorder by the National Institute of Health and Clinical Excellence (2018) guidelines as well as numerous international guidelines (APA, 2017; ISTSS, 2019). In this paper, we present how to treat PTSD arising from traumatic bereavement, where the patient’s worst fearful expectations have come true, resulting in the death of someone close to them. A significant component of the treatment involves imagery transformation and this will be covered with the procedure of memory updating.

### Ehlers and Clark’s cognitive model of PTSD

**Figure 1.**
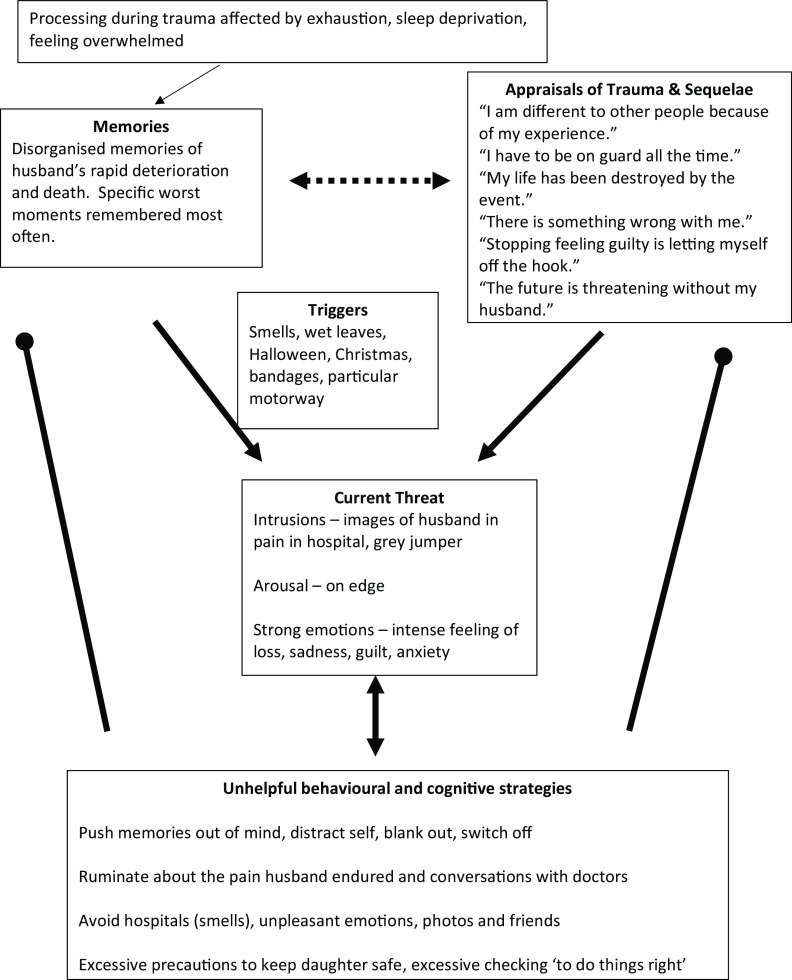
Cognitive model of PTSD (Ehlers and Clark, 2000) illustrating a patient’s PTSD arising from the traumatic death of her husband. Pointed arrows stand for ‘leads to’; dashed arrows stand for influences, and round arrows stand for ‘prevents change in’.

Cognitive therapy is rooted in the idea that whilst people may face difficult times, it is the meaning they make of them that matters. Whether PTSD arises as a result of the sudden death of a loved one or another traumatic event, a significant focus of CT-PTSD will be updating the meaning the patient attributes to their trauma, particularly the worst moments. Ehlers and Clark (2000) propose three core processes that keep PTSD in place and reinforce a sense of external or internal threat in the present. The first process encapsulates the disjointed nature of trauma memories, which are easily triggered and poorly integrated with the patient’s autobiographical memories so that they are retrieved without a context, as if the event was happening now. The second process relates to the meanings the patient makes of their trauma and what has happened since, captured in their idiosyncratic appraisals of the trauma, its worst moments and subsequent events. Finally the patient may embrace strategies, such as suppression of memories, dwelling about parts of the trauma, and safety-seeking behaviours including over-checking for danger or social withdrawal, in attempts to cope with their difficult symptoms, feelings and memories, yet which typically maintain them. The model in Fig. 1 illustrates PTSD arising from a traumatic bereavement for a patient, Faye, who lost her husband due to a medical mis-diagnosis.

In this example, Faye’s trauma memories were from the last few months of her husband’s life during which he had surgery that went wrong for a delayed cancer diagnosis, followed by his death a few weeks later. When recounting the trauma, Faye started with the worst moments, such as recounting the surgery then turning to the moment her husband received his diagnosis and then recounting his last moments. Her trauma narrative was out of sequence and did not include information that she had now, such as ‘he is no longer suffering’. Her trauma memory was easily triggered by matching cues in the environment, such as the smell of alcohol wipes used to clean his post-surgery incisions, hand sanitiser, wet leaves similar to those in the parking lot during her hospital visits to see him, bandages, advertisements for Halloween since he was rushed to hospital in the days before Halloween, a particular motorway which she drove frequently to visit him, and Christmas because he died shortly afterwards. She often experienced images of the worst moments: her husband suffering in pain in hospital and his grey jumper which he was wearing when they received his diagnosis. When the memory or images were in mind, she experienced the anxiety she had at the time as well as strong feelings of loss. She tried hard to push the images and memories away, to distract herself, blank out with a glass of wine or switch off. The images triggered episodes of rumination in which she would question why she did not push for a second opinion when the initial diagnosis was stress. She would replay conversations with the doctors in her mind over and over. Faye avoided hospitals, she would not visit friends or other family members, such as her mum, in hospital and she would cancel her own hospital appointments. In response to feeling sad, she would turn to work, keeping busy and trying hard to avoid thinking about what happened. In response to the experiences of intrusive memories and comments from friends as well as her GP that she should have moved on by now, Faye believed there must be something wrong with her and that she was different from other people. She believed that the loss of her husband had destroyed her life, that the future was threatening without him, that she had to be on guard all the time to protect her daughter and to check that things were being done ‘right’. She believed that if she stopped feeling guilty, she would be letting herself off the hook.

For this case and other trauma leading to post-traumatic stress, CT-PTSD works to elaborate the trauma memories, update their meanings and discriminate triggers to reduce re-experiencing symptoms. The treatment will identify and update maintaining appraisals as well as help the patient to give up cognitive and behavioural strategies which maintain the appraisals and disjointed memories, and thus the sense of current threat; whilst at the same time helping the patient to reclaim or rebuild their life with activities that give a sense of enjoyment and purpose. **A core focus of CT-PTSD when applied to bereavement trauma is to reduce the sense that the deceased is still suffering or not at peace and create a sense of continuity in the present with what has been lost in the past.**

PTSD following bereavement trauma will include a number of re-experiencing symptoms, such as intrusive memories of the loved one’s suffering or death, avoidance of reminders of the suffering or traumatic death, negative alterations in cognition and mood and a number of hyperarousal symptoms, such as difficulty sleeping, feeling more irritable, on edge, engaging in risky behaviours or feeling significantly more alert and on guard. The core element of prolonged grief or complex grief is persistent yearning for the deceased. The patient may experience pre-occupation with the circumstances of the death, difﬁculty accepting the death, feelings of loss of a part of oneself, anger about the loss, guilt or blame regarding the death, or difﬁculty engaging with new social or other activities due to the loss. Please see Duffy and Wild (2023) for a detailed description of the overlapping and distinguishing symptoms of persistent bereavement disorder and PTSD.

## Treatment components

CT-PTSD will follow a similar course when treating PTSD linked to traumatic loss or PTSD related to other events. Below we describe the core treatment components and how they are adapted for PTSD associated with bereavement trauma. The structure of the treatment will be similar to PTSD linked to trauma where there is no loss of a significant other, and about 10–12 sessions will be offered. For videos on how to deliver the treatment, please see www.oxcadatresources.com and Wild *et al*. (2020) for a guide on delivering the treatment remotely.

### Considerations specific to traumatic bereavement

With traumatic bereavement, it is important that the therapist empathises, acknowledges the loss and the importance of the relationship, and does not rush into cognitive restructuring of worst meanings. Many patients who have experienced loss will also have had the experience of being unable to talk about their loss or loved one with friends or family after the initial months. This may be a result of believing they are a burden or being told that they should be moving on. It is important that the therapist encourages the patient to speak about their relationship with the deceased in session as well as share details about the person who died, and this may involve showing photos of the deceased in therapy. Sometimes patients are distressed that they felt a sense of relief after loss trauma, which is common when they have looked after an ill loved one. The therapist will want to normalise the emotions the patient is feeling, whether this is grief, anger, relief or any combination thereof. In the course of therapy, the therapist will elicit the meanings of death for the patient and this can be facilitated by using weekly questionnaires, discussed below. Common meanings relate to self-blame, regrets about things that were said or not said, and life no longer having meaning. The therapist will work with the patient to identify updates for these meanings before detailed memory work. Whilst bereavement trauma can shatter a patient’s spiritual beliefs, this is not always the case and the therapist will need to discuss the patient’s beliefs about an afterlife and cultural beliefs about death before updating. When working with patients who may be low in mood or suffer from co-morbid depression, the therapist needs to be mindful of risk and to intervene accordingly. Should risk become the primary problem, then the therapist will need to follow service-specific guidelines, which will include contacting the GP and may include referral to crisis teams or A&E. Finally, an important task of therapy is to help the patient to shift their focus from loss to what has not been lost, from a focus on their loved one being gone to considering how they may take their loved one forward in an abstract, meaningful way. This concept, *intangible continuity*, is about moving forward with the meaning of the loved one, which the patient can access at any time through imagery, a personal value or quality, behaviour, or activity. Intangible continuity is a component of memory updating for bereavement trauma and examples are given in the ‘*Memory updating and imagery transformation*’ section below.

### Individualised case formulation

**Figure 2.**
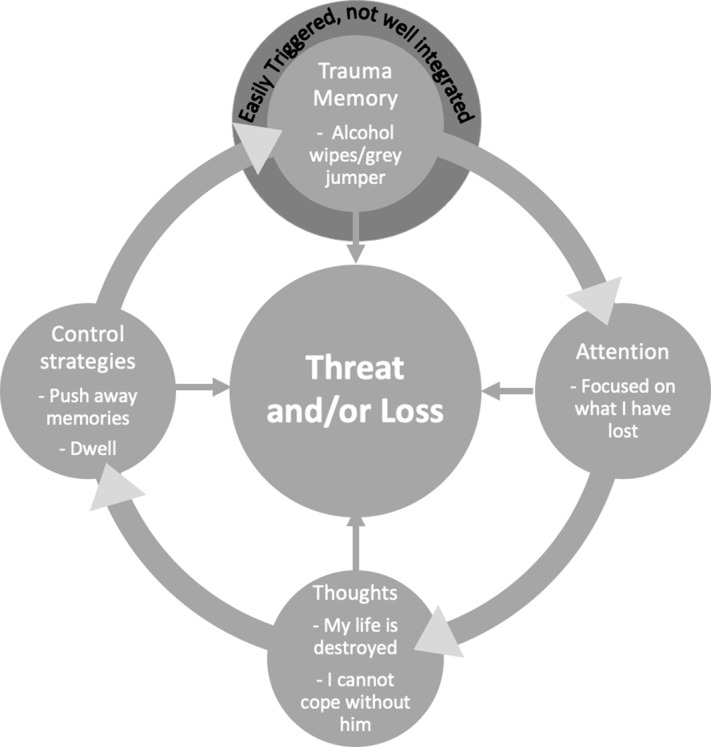
A maintenance cycle shared with the client showing the processes treatment will target.

The therapist will assess the current problem, agree the patient’s goals, and develop a case formulation which guides treatment and may be elaborated as therapy progresses. The Ehlers and Clark (2000) cognitive model of PTSD illustrated with a clinical example in Fig. 1 above would typically not be shared in detail with the client. Instead, the therapist will draw a broad cycle with the patient, encapsulating the processes and strategies that maintain PTSD and strong feelings of loss, which helps to illustrate the targets for treatment. Symptom measures, such as the PTSD Checklist for DSM-5 (PCL; Weathers *et al*., 2013) and the Prolonged Grief Scale (PG-13; Prigerson *et al*., 2021), process measures, such as the Posttraumatic Cognitions Inventory (PTCI, short-version; Kleim *et al*., 2013), the Safety Behaviours Questionnaire (SBQ; Dunmore *et al*., 2001) and the Responses to Intrusions questionnaire (Clohessy and Ehlers, 1999) help to inform the therapist’s formulation. As therapy progresses, the measures help to gauge whether interventions are working as expected and to guide what adjustments may need to be made. Figure 2 depicts the cycle shared with the client, Faye, to illustrate the maintaining processes to be targeted in therapy.

In this cycle, the traumatic memories are easily triggered as is the case in PTSD. When triggered, the patient feels anxious and tries to push the memories from her mind, a strategy which typically keeps them there for longer and maintains her sense of threat. Whilst in mind, the patient’s attention turns to what she has lost and she has appraisals about her life being destroyed by the event, the future as threatening without her husband, needing to be on guard all the time to keep her daughter safe, and being different from other people as a result of what happened. These thoughts keep her focused on loss. The patient pushes the memory out of mind or tries to make sense of what happened which causes her to over-think the trauma without accessing new information, a process which keeps her focused on loss and keeps the trauma memory in mind unable to be more fully integrated with her other life experiences. Thus, treatment will help to shift her focus and work with the thoughts, strategies and the memory which are keeping her PTSD in place so that she may move forward with loss rather than feeling stuck in it.

### Rebuilding life activities

One of the goals of CT-PTSD is to help patients to be able to live the lives they would like to live. This is achieved through symptom reduction as well as by guiding the patient to engage with reclaiming or rebuilding life activities that bring a sense of purpose and are enjoyable. When trauma has resulted in objective permanent changes, such as through the death of a loved one or a permanent physical injury, CT-PTSD typically encourages patients to engage in activities to rebuild their life. Loss trauma dramatically changes a patient’s life and it is not always possible to return to a way of life the patient may have had before the trauma. The first step in planning rebuilding life activities is to focus on ones that include self-care, social connections, enjoyment, or give a sense of meaning or satisfaction. The activities may be ones the patient used to do that they could reclaim, activities that can replace ones they used to do, and new activities they would like to start. The therapist and patient will consider important domains in the patient’s life, such as exercise, self-care, interests and hobbies, relationships, work, studies and other interests and then identify activities that they could try that fit with these domains. The guiding principle with rebuilding life activities is to plan achievable activities from the first session then onwards throughout therapy. The activities can be short and gentle and should fit with the patient’s goals for treatment.

*For example, Faye kept herself busy after the death of her husband. She worked full-time and cared for her daughter. One of her goals for treatment was to feel calmer and more relaxed. The initial rebuilding life activity that she identified with her therapist that would fit with this goal was experimenting with taking one 5-minute tea break a day. Faye was able to fit this in and found that she enjoyed the short break without distractions, describing it as ‘quite nice’.*

Some of these activities can also be undertaken by the bereaved person as a means of taking the memory of the deceased forward with them, and are more likely to be introduced when the patient has begun to work on shifting focus from what has been lost to moving forward with loss. For example, a father decided to start cycling partly to honour the memory of his son who was a cycling enthusiast. He had a badge with his son’s picture on the bike frame and when he would start to feel like taking it easy he could imagine his son encouraging him saying ‘Go on Dad, push yourself’.

When planning rebuilding life activities, the therapist will elicit and address any inhibiting thoughts the patient may have to carrying them out. Table 1 shows common inhibiting thoughts after loss trauma and core messages to convey when addressing them.

**Table 1.** Inhibiting thoughts to engaging in rebuilding life activities and how to address them

Inhibiting thought	Core message to convey	Therapeutic interventions
‘It’s not important. Everything seems insignificant’.	It is important to convey that such feelings are normal after trauma and that moving towards goals can start with a small step. A small step may even be rewarding and lead to valuable information about the planned activity. The therapist could suggest that the client experiments with a small step then re-assess how they feel, making a decision to keep going with the activity or to stop.	Normalisation Psychoeducation Collaboration Behavioural experiments
‘It’s disloyal. I don’t deserve to be enjoying myself’.	It is normal after the loss of a loved one to feel that any sense of enjoyment is disloyal. The therapist may find it helpful to gently ask the patient if their loved one would agree that trying to rebuild their lives was disloyal. What would their loved one want for them? Would they want them to stay stuck or to try an activity that could give them a sense of enjoyment even if only for a few minutes a day? The therapist may also ask: ‘If it had been you who had died, would you say your loved one was being disloyal? What would you want for them?’	Normalisation Imaginal conversation with loved one about moving forward with life.
‘It won’t be the same without them’.	Engaging again in activities the patient enjoyed with their loved one is unlikely to feel the same. It will be important to acknowledge this. The patient may benefit from setting up a behavioural experiment where they shift their focus of attention during the activity. It is common for patients to try previously shared activities whilst focused on their loved one not being there, which intensifies the sense of loss. The patient may experiment with trying the once shared activity and focusing their full attention on what they can see and hear rather than on the absence of their loved one. They may also like to try the idea of nurturing their loved one’s memory through doing the things they enjoyed together and on ‘their behalf’.	Normalisation Behavioural experiments
‘I have lost too much. Life is meaningless now’.	After the loss of a loved one, it is normal to feel as though too much has been lost to contemplate trying an activity that might bring relief or enjoyment. The therapist may find it helpful to gently ask the patient what they predict would happen if they were to try an activity like a tea break for a few minutes and then set up a behavioural experiment to discover if their worst fears occur. It can be helpful later in therapy to guide the patient to identify what has not been lost. Focusing on what has not been lost can improve mood and make it more manageable to plan new rebuilding life activities.	Normalisation Behavioural experiments
‘I’m different to other people. No one will be able to relate to me’.	As trauma can make people feel as though they are different from others, the therapist may want to convey the idea that whilst our experiences may differ, our values are likely to be similar and it is still possible to find common ground. People often equate the intensity of their bereavement with the sense of being different from others: the stronger the feelings of loss, the stronger the belief that they are different from other people. Our feelings are actually more likely to be similar to others but at different times. Most people have experienced bereavement and can empathise with how painful it is. It is also true that connecting with others is important in recovering from loss-related trauma where there is grief. The therapist may want to plan a behavioural experiment where the patient tests predictions about what would happen and how others would respond if they were to engage in a short activity with other people.	Psychoeducation Behavioural experiments

### Addressing core cognitive themes

With loss trauma, the patient’s worst fears did happen and there may be little information they have since discovered that contradicts the meaning of their worst moments. With trauma where there is no loss of life, the patient’s worst fears may not have happened in the way they thought at the time and new information may be more readily available to update worst moments. Cognitive themes therefore for bereavement trauma will typically involve permanent change, loss and guilt and work on these themes will precede imaginal reliving and updating, which will almost always include imagery transformation. Below we describe the most common cognitive themes associated with loss trauma and the therapeutic interventions used in CT-PTSD to address them. Use of weekly questionnaires, such as the Post-Traumatic Cognitions Inventory (PTCI; Foa *et al*., 1999), help to assess and track changes in trauma-related appraisals linked to feelings of guilt, anger, over-generalised fear, shame, permanent change, alienation or persistent degradation. Please see www.oxcadatresources.com for commonly used symptom and process measures in the treatment of PTSD, videos on how to use them and how to work with a range of cognitive themes.

#### Meanings of loss

Understandably traumatic bereavement leads to thoughts and feelings of loss. A key aim of therapy is to differentiate beliefs that are normal after bereavement and that generate sadness such as ‘I have lost my loved one and will miss her dearly’ from idiosyncratic added negative meanings that generate pathological grief such as ‘I have lost everything’, ‘I am permanently changed’ and ‘I am forever alone’. Such appraisals, whilst maintaining PTSD, also keep traumatic grief in place and can be inhibitors to the patient engaging in rebuilding life activities. A few sessions into treatment when the patient may have benefited from some rebuilding life activities, the therapist may gently guide the patient to think about what has not been lost and what the deceased would want for them. The therapist will be working to shift the focus from loss to moving forward with loss. The therapist may use surveys to help normalise the patient’s feelings of loneliness and isolation as well as to generate compassionate responses from other people, which may help the patient to take steps to engage in social activities. For further discussion on how, when and why to use surveys in CBT, please see Murray *et al*. (2022). The therapist will often use imagery transformation to create a sense of continuity with the meaning of the person who died, focusing on how the patient can take the loved one forward with them. The therapist may summarise core learning on a flashcard. Updating information, such as what the patient has not lost and how they are taking their loved one forward with them, will be brought back into the trauma memory when the patient and therapist work together on the memory.

*Jasmine’s son died in a road traffic accident. She had two surviving children. She believed she had lost everything and that she was all alone, that no-one would understand or want to socialise with her, that she was burdensome and that she would experience zero joy from being around other people. The therapist created a survey with Jasmine and then a behavioural experiment. See Table 2 for an example of the completed survey with one respondent; see Table 3 for the experiment the therapist planned with Jasmine.*

**Table 2.** A respondent’s completed survey

**Survey** A mother of three lost her son in a road traffic accident three years ago. She no longer wants to go out, believing that she is a burden and that no-one would want to socialise with her. She believes that seeing other people would bring her zero joy. How much, out of 100%, do you think this woman is a burden because of her experience? *Bereavement is so tough and it’s common to feel like a burden because the feelings are burdensome for the person who has lost someone they love. I empathise. I would not find this person a burden. 0%* If you met her at a social occasion, would you want to socialise with her? *I would definitely want to socialise with her. Our experiences make us who we are.* If this were a friend of yours, what would you say to her? *I would hug her and say how sorry I am at what she has gone through, I do understand how devastating it is and how there will be times it will be tough to get out of bed in the morning, let alone try to meet up with people. I would encourage her to take small steps and to be kind to herself, moving forward with this grief takes one step at a time. Over time, moments of joy will punctuate her grief after lots of small steps. I’d ask her how I could help and what I could do that might make things more manageable for her like cooking dinner or taking her other two children out with mine whenever she would like.*

**Table 3:** Jasmine’s behavioural experiment

Situation	Predictions	Experiment	Outcome	Learning
Going for coffee with Fatma, Zaheera and Cindy	I will be a burden 100%. I will know this because my friends will want to leave after 20 minutes or they will make efforts to change the conversation They will tell me I should think about moving on 100%	Go for coffee, be honest about how I am feeling when asked without apologising	Had coffee, only intended to stay for an hour but we chatted for almost two and a half hours. I did cry and they were so great. Zaheera held my hand. Although I felt like a burden my friends did not treat me like one No one told me I should be moving on	It was hard to go out. I’m glad I did, I do feel better for it and I wouldn’t have discovered this if I had avoided it. Maybe seeing some friends on occasion is good for me I am a burden 50% My friends think I should move on 50%

The therapist created a flashcard for Jasmine with the learning from the behavioural experiment and the survey (see Table 4). They added to the flashcard what she thought her son would want for her, as well as how she would take her son forward with her. When Jasmine spotted the urge to avoid seeing friends or to try other rebuilding life activities, she would read her flashcard then give the activity a go.

**Table 4.** Jasmine’s Flashcard

**My Flashcard** Samuel would want me to smile. He would want me to see my friends. I have discovered that although I find my feelings burdensome, no-one treats me like I am a burden. I have tiny moments of relief when I see my friends and I will build on this by trying more activities. I have not lost Jerome or my daughter, Latitia. Samuel was pure love and innocence, he meant the world to me. I take him forward when I feel love, he is a part of that, a part of me when I’m feeling loved. I take his innocence forward in my values of honesty and good-naturedness with my family, my colleagues and my friends, this value that captures Samuel is with me, a part of me.

Updating information is then brought back into the trauma memory at the point in therapy when the therapist and patient are conducting memory work. This procedure is discussed in the next section, ‘*Memory updating and imagery transformation*’. Here we see Jasmine’s updated memory related to the moment of Samuel’s death:

‘*Samuel sets off for school on his sister’s hand-me-down bike. He’s not gone two minutes when I hear a colossal screeching of brakes. I run out and he is in the road. Laying in the road! I cradle over his little body weeping, Sam, Samuel. I think paramedics arrive quickly they take me to the side and start resuscitating him, I see a paramedic’s shoulders shake and he shakes his head. The other paramedic walks over to me, head down, he puts his hand on my shoulder and says they did everything they could. The driver is questioned by the police. I am stunned, devastated, it doesn’t feel real. The police officer calls my mother. She breaks down when she arrives and we hold each other. They take Samuel’s body for a post-mortem. He is gone. Just like that. My beautiful boy gone forever. I am forever without him, alone. I am devastated. I have lost everything. I have nothing…’* [Pause, therapist prompts ‘What are you feeling, Jasmine?] *‘I feel devastated, alone, forever alone and so deeply sad*.’ [Therapist prompts: ‘You’re doing a great job, Jasmine, stay with the feeling…’] ‘You feel devastated, alone, deeply sad…[pause] and when you’re ready I’d like you to bring in the new information, call to mind what we know now.’

*‘… I now know I’m not alone. I know that Jerome and Latitia are healthy, they are safe, they are with me, they need me. I have not lost them. I have a beautiful photo of them on my phone cuddling on our new sofa. I now know that to me, Samuel means pure love, innocence. I take him forward in the innocence I see in Jerome and Latitia, in the innocence I hear in morning birds waking the day, in the innocence of my husband when he tries to help with the cooking, carefully cutting onions but leaving the skin on … I take Samuel forward with me when I feel love, he is there a part of me, I take him forward in my innocent honesty, my values that I share with my family, my colleagues, my friends. This value that captures Samuel is with me every day, a part of me, shapes me*.’

#### Guilt

No matter how much the patient has cared for or supported the person who died, they often feel guilty for being unable to prevent their death or suffering. In CT-PTSD, we address guilt by widening their perspective and exploring all the other factors that led to the death. The discussion is summarised with a responsibility pie chart, looking at all the factors that might be responsible for the death of the patient’s loved one. We ask the patient to estimate their percentage of responsibility only after considering the contribution of all other factors. Importantly, the therapist will guide the patient to focus on all that they did that was helpful during the life of their loved one, not just through illness or in the moments before their death. The aim is to enable the patient to take a step back and evaluate their behaviour in the context of the person’s whole life as well as to gain a more objective evaluation of what they did that was helpful around their death, to shift the focus from ‘what I didn’t do’ to ‘everything that I did do’. Psychoeducation, including input from relevant experts or reliable online sources, as well as surveys, can be instrumental in helping to update a patient’s appraisals about the effect of the behaviour they believe shortened their loved one’s life or lengthened their suffering.

*Tanya’s husband, Derek, was living with obesity. He died suddenly of a blood clot. Tanya believed that she could have prevented Derek’s death if she had acted faster and made her husband seek medical help two days earlier when he was starting to feel lethargic. Below are the questions the patient and therapist put together for a medical person to answer to gain information that might be used to update Tanya’s responsibility appraisal. The therapist then contacted a paramedic she knew and asked if they would complete the survey anonymously (see Table 5).*

**Table 5.** Example survey completed by a paramedic

**Survey** A man living with obesity had an undetected DVT. He died whilst the ambulance was pulling into the driveway of his house where he lived with his wife. What do paramedics do to assess for DVT? *Paramedics would rule out or rule in DVT depending on the following factors – if these are found, then the likelihood of DVT is high:* *Patient has active cancer and treatment* *Paralysis or recent plaster immobilisation of the lower extremities* *Recently bedridden for 3 days or major surgery within the last 12 months* *Localised tenderness along the deep venous system* *Entire leg swollen* *Calf swelling at 3 cm larger than asymptomatic leg* *Pitting oedema to the symptomatic leg* *Collateral superficial veins (non-varicose)* *Previously documented DVT* *Heart rate more than 100* Roughly what would you say the survival rates are for people who have DVT? *Survival rates are low.* If someone has a DVT, does getting them to the hospital as soon as possible help survival rates? *It’s important to get them to hospital but survival depends on a lot of factors. Certain factors increase chance of poor outcome, such as living with obesity, poor mobility, swelling, recent surgery. We transport a lot of patients to hospital with DVT but that doesn’t mean they survive. I know of a few patients who were admitted with DVT and did not survive. It’s obviously important to act quickly but it does not guarantee survival.*

**Figure 3.**
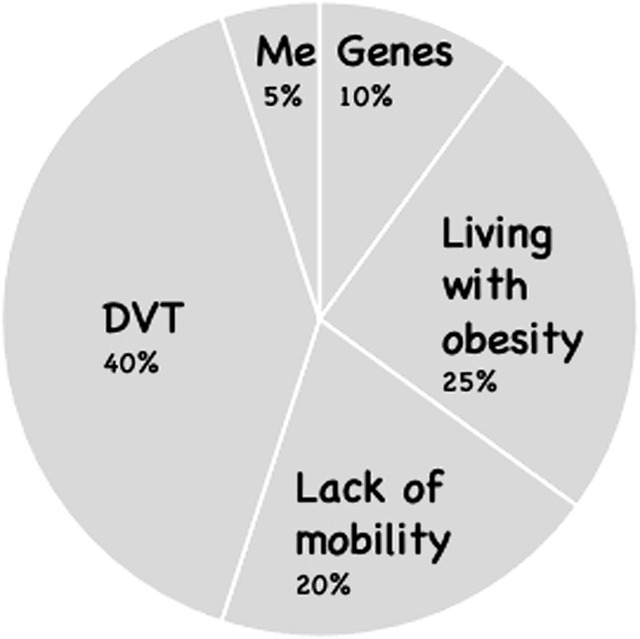
Responsibility pie chart.

Prior to the survey, Tanya believed she was 100% responsible for the death of her husband. After they reviewed the results together, the therapist created a pie chart with Tanya, first taking account of all the contributing factors to her husband’s death and then asking her to rate her contribution. Figure 3 illustrates the pie chart and her sense of responsibility (now 5%) after considering other factors.

If, after cognitive restructuring, there is little change in the patient’s sense of responsibility, it is possible there is a head–heart gap where the patient may understand logically that they are not responsible for their loved one’s death, yet continue to feel that they are. The patient may benefit from having a conversation in imagery, following the principles described in the ‘*Addressing complexities*’ section below. A conversation in imagery can help the patient to experience the compassion their loved one may have felt for them and to hear a kinder perspective, likely the one their loved one would have held, which is often associated with an experiential shift in their sense of responsibility. Perhaps this is unsurprising given that research suggests that an experience in imagery may be akin to a real experience (Epstein, 1994).

#### Perceived permanent change

After loss trauma, many aspects of a patient’s life will have permanently changed and the therapist acknowledges this with empathy. Yet, the patient’s negative appraisals may be over-generalised: they may experience themselves as permanently changed as a person, to have lost everything worthwhile in their life, believe that their life is now meaningless or that they are all alone in the world, unable to have a connection with anyone other than the person they lost. Of course, our experiences do shape us, but what seems to remain constant are our values. Helping patients to discover that whilst our experiences will differ from one another, our values are more likely to be similar and can help patients to approach rather than avoid social encounters and other rebuilding life activities. Moving forward with loss will often mean creating new routines, finding new joys and relating to other people. As described above, it also means creating a new relationship with the person who died by thinking about them differently, spotting their meaning and connecting to their meaning in an abstract way. Therapy will also help the patient to spot what has not been lost so that the patient may feel more integrated with their entire life’s experiences rather than defined by the loss trauma.

When a patient has been socially withdrawn for a long period, such avoidance is often linked to appraisals such as ‘I am changed for the worst and other people will see me as different or strange’. Behavioural experiments can be used to test the validity of such beliefs. The therapist may need to prepare the patient in setting up such experiments that some people may find it awkward and difficult to speak to a person who has been bereaved, and it is important to avoid interpreting others’ behaviour as confirmation of the beliefs about ‘a changed self’. As an example, a mother who had lost a child found that one woman seemed a bit distant during a behavioural experiment but in therapy was able to clarify that she was an anxious mother who self-referenced the patient’s loss and could not cope with the thought of losing her own child who already had a chronic health condition. Another problem may be that in first encounters with some people after bereavement, there may be a ‘natural’ inquisitiveness to ask and over-focus on the death of the loved one, so role plays can be helpful to prepare responses, accept empathic remarks and then steer the conversation onto other subjects.

#### Anger

When working with appraisals linked to anger, psychoeducation about the reasons for other people’s behaviour may be important (e.g. why certain types of cancer are misdiagnosed) as well as considering the advantages and disadvantages of holding onto anger. It can be helpful for the patient to write an ‘anger letter’, which they do not send, to the person they are angry with, spelling out why they are angry and what they want the person who harmed their loved one to know. When the patient’s loved one suffered in hospital or received poor care, they may decide to write a letter through the appropriate process to raise the concern and makes suggestions on how care could be improved, which may help reduce the likelihood of someone else experiencing something similar. This can help the patient to use their anger in a constructive way. The therapist may also conduct a survey to gather information to normalise how the patient is feeling and support them to let go of anger. In circumstances where the loved one has been killed by intentional acts of violence such as wars and conflicts, anger can be prominent and strong. In such cases it is crucial that the therapist empathises with such anger, described by one patient as ‘righteous anger’. It is important to discuss the legitimacy of such anger as an initial response whilst recognising the effects of becoming entrapped in hatred and hostility that can deteriorate into bitterness and thoughts of vengeance. In some cases bereaved parents will initiate campaigns for justice which are important actions provided the lives of the bereaved are not consumed by such campaigns at the expense of caring for other surviving family members. There are a number of helpful questions to address these issues, such as: ‘Who wins with anger?’, ‘What will the effect be on your remaining family members if you stay angry?’ and ‘10 years from now looking back, how will you like your children and grandchildren to remember their father or grandfather – as a constantly angry man or a man who was able to relate to them despite his loss?’.

#### My loved one is still suffering or cannot find peace

These types of meaning usually have two sources: impressions from the trauma that are re-experienced (e.g. the loved person in pain or looking frightened; appearance of their face after death) and appraisals about the circumstances of the death or funeral that violate cultural conventions (e.g. not able to prepare for death with culturally appropriate rituals; loved one unable to move on to afterlife; expectation that all people feel extremely frightened during death; dying alone without support and perception of feeling unloved; undignified death was shameful; body treated disrespectfully; mistakes in funeral procedures). Regrets about the patient’s last interactions with the person can also contribute to these appraisals. These are addressed by discussion of features of trauma memories (e.g. these memories appear as if they are happening ‘now’, so each time you have this memory of your husband looking frightened it feels as if he is frightened now), work on triggers of these memories, and the updating memories procedure described below, including imagery transformation.

### Memory updating and imagery transformation

Memory updating is a procedure used in CT-PTSD to elaborate the trauma memory, identify information that updates the worst meanings, and then link new information to the respective worst moment in memory to make these less threatening. In CT-PTSD for traumatic bereavement, imagery transformation is typically incorporated with the memory updating procedures, both of which are described below.

Updating trauma memories in CT-PTSD involves three steps:

- *Identify threatening personal meanings*. First the therapist and patient will *identify worst meanings*. Imaginal reliving or narrative writing and discussion of the content of intrusive memories will help the therapist and patient to identify the moments during the trauma associated with the most distress and sense of ‘nowness’ (‘hot spots’; Foa and Rothbaum, 1998) and the worst meanings of these moments.
- *Identify updating information*. The therapist and patient will work together to identify updating information that helps to make the meanings of the worst moments (‘hot spots’) less threatening and thus less distressing. For traumatic bereavement, this information may be discovered through work on cognitive themes. Cognitive restructuring techniques can be used to update appraisals linked to guilt, shame, anger, perceived permanent change and loss. These include Socratic questioning, surveys, pie charts, behavioural experiments, positive data logs and use of imagery techniques.
- *Link the new information to the relevant moment in memory.* Once the therapist and patient have discovered information that updates the patient’s meanings for a particular hot spot, the therapist guides the patient to hold the hot spot in mind whilst recalling or experientially taking in the new information. This can be done verbally (e.g. ‘I now know…’) whilst bringing the hot spot to mind in reliving or when reading through the hot spot and the updates in a narrative. Where possible, the new information can be strengthened or demonstrated experientially through sensations or movement that makes the new meanings salient, including with imagery. In traumatic bereavement, the patient is often encouraged to call to mind an image that represents the updating information, such as that the loved one is no longer suffering. Imagery may also be used to show the patient how they are taking their loved one forward with them in an abstract, meaningful way.

#### Imagery transformation

**Figure 4.**
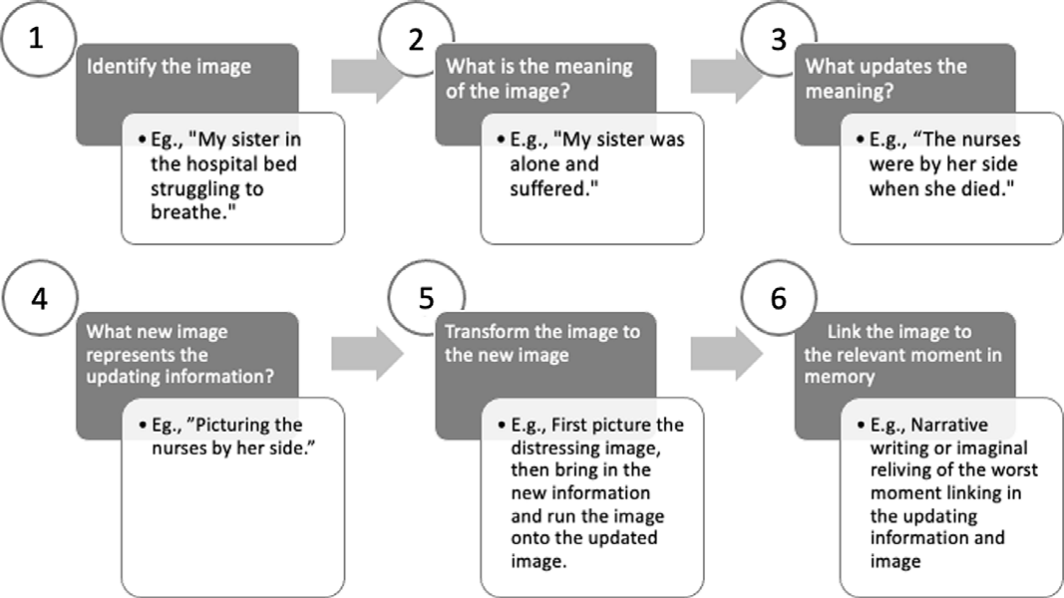
Steps to transforming images of loss with CT-PTSD.

With traumatic bereavement, the patient often experiences distressing images of the person who died. The images may be snapshots of memories of worst moments or perceived suffering. The images maintain distress because they link meanings related to the deceased’s continued suffering (e.g. image of my wife’s face in distress when she died from a catastrophic brain haemorrhage, meaning ‘My wife is dead. She is suffering forever because of how she died’) or loss (e.g. image of my daughter’s face as her life faded away, meaning ‘I’ve lost my daughter. I’ve lost the rest of my life’) or guilt (e.g. image of my boyfriend on the road after a cardiac arrest, meaning ‘I should have known how to do CPR; he died because of me’). Such meanings are difficult to update in the context of repeated re-experiencing. The persistent images contribute to difficulties in recalling memories of the deceased before they died and as such, prevent the loss memory from being more fully integrated with other autobiographical memories from their relationship or relevant information (e.g. their pain was limited by medication; they were unconscious). When the images are in mind, patients may feel as though they are losing their loved one all over again. Transforming the distressing images can help to update the trauma memory and linked appraisals. There are six steps to transforming images of loss summarised in Fig. 4.

*1. Identify the image.* The first step is to identify the image. Patients may disclose in assessment or in discussion of questionnaires, notably the PTSD Checklist for DSM-5 (PCL-5; Weathers *et al*., 2013), that they are distressed by images of their loved one suffering. This gives clues early on about images the therapist is likely to be working with in treatment. As intrusive imagery is often linked to particular moments from the trauma memory, the therapist can prompt the patient to describe the main unwanted memories that repeatedly come to mind and ask if they experience an image of any part of the memory that is distressing, vivid and recurrent. When the patient has beliefs related to their loved one’s continued suffering it is important to ask them to describe their perception of their loved one’s experience of death beyond the point of dying. Patients may experience images of worst fears, such as their loved one being buried sentient and aware, which did not happen. We strongly recommend that the therapist ask how distressing the image is on a scale of 0 (low) to 100 (very distressing) and how ‘now’ it feels from 0 (like a memory from the past) to 100 (happening in the here and now).

*2. Elicit the meaning of the image.* The next step is to elicit the worst meaning of the image. The worst meaning extends beyond the recognition that the loved one died and carries substantial emotional pain. We have found that worst meanings linked to images of suffering usually relate to perceptions of pain that were perceived to be undeserved or preventable, of their loved one feeling deserted or of their loved one’s fear of dying or of perceived suffering as continuing in the present, or responsibility for failing to prevent suffering, including not being with the loved one when they died. Exploration of these meanings may require a discussion of the patient’s beliefs about an afterlife and cultural or religious norms about dying (e.g. rituals and rules that ensure a successful transition to the afterlife). Table 2 gives examples of common meanings associated with distressing images linked to loss.

*3. Consider what information the patient may have now that updates the worst meaning.* The third step is to work together to update the worst meanings. New information can emerge through imaginal reliving or narrative writing of the traumatic loss memory. Most often guided discovery and cognitive therapy tools can address cognitive themes (outlined in the ‘*Addressing core cognitive themes*’ section above) and elicit updating information. Table 6 shows common appraisals and CT-PTSD tools to elicit new information.

**Table 6.** Common appraisals and the CT-PTSD tools used to elicit updating information

Worst meaning relates to	Example appraisals	CT-PTSD tools used to elicit updating information
Suffering	My loved one suffered enormously They are still suffering	Narrative writing of the traumatic loss memory Imaginal reliving Psychoeducation Guided discovery – is the suffering over now? Would the patient agree that their loved one is no longer suffering? Speaking to experts – e.g. how commonly are people buried whilst still alive? Researching people’s near death experiences to ascertain whether they fit with the patient’s worst fears
Dying alone	They died alone They didn’t deserve this Their suffering was worse because they died alone	Narrative writing Guided discovery – were they alone? Information about drugs if given pain meds. Consider level of consciousness at the time Surveys with or speak to experts
Guilt about death	I didn’t prevent their suffering I could have done more They didn’t deserve this The doctors let them down I let them down	Responsibility pie charts. What did the patient do that was helpful throughout the relationship? Surveys
Guilt about things said/not said	They carried my last words to their death, this is how they remember me	Review of relationship, what did the patient do to support them or show them that they cared? Conversation with loved one in imagery
Perceived permanent change to the self and to life in general	Life will never be the same I will never be the same Life has no meaning	What has not been lost? Shift the focus – how can I take them with me? How did they influence me? What qualities did I love about them, how can I connect with those qualities today? How can I take them forward in my actions, an image, a personal value or quality, behaviour or activity that captures their meaning?
Loss of meaning	I am alone now Life has no meaning I have lost everything	Shift the focus. How can I take them forwards? Conversations in imagery What has not been lost?
Pre-occupation with injustice/anger	The doctors/perpetrator/other person robbed them of their life They would still be alive if it wasn’t for the other person	Imaginal reliving or narrative writing Guided discovery Anger letter Surveys Advantages and disadvantages of letting go of anger Empathising with understandable anger and distinguishing from hostility, hatred, bitterness and vengeance
Degradation/shame	My loved one died whilst engaged as a combatant in conflict. I have no right to the empathy of others. I deserve to suffer until I too die	Surveys Conversation with loved one in imagery Behavioural experiments
Violation of cultural norms	They will not find peace in the afterlife. They are still suffering	Narrative writing of the traumatic loss memory Imaginal reliving Guided discovery – is the suffering over now? Would the patient agree that their loved one is no longer suffering? Imagery transformation Surveys with or speak to experts

Below is an example of new information that came to light after narrative writing of the traumatic loss memory.

*Samira lost her sister to cancer during the COVID-19 pandemic. Samira had recurrent images of her sister dying alone and struggling to breathe. The worst meaning of the image was Samira’s belief that her sister suffered enormously and died alone. Imaginal reliving of the trauma had revealed that Samira’s sister was holding the bracelet Samira had given her and they’d shared over the years in her hand when she died. The nurse who telephoned Samira when her sister passed away had said she and another nurse were at her bedside when she died.*

In this example, narrative writing revealed that two nurses were beside Samira’s sister when she died. The therapist helped Samira to update the worst meaning from ‘*She suffered enormously and died alone*’ to ‘*She held my bracelet when she died, wrapping my love for her in her fingertips, captured in that bracelet. The nurses, Stella and Lizanne, were with her when she passed away. She died in the loving thoughtfulness of kind-hearted nurses with a bracelet that caught our connection. She did not die alone. She is no longer suffering.*’

Typically information that the loved one is no longer suffering is included in updating information. We also often include how the patient would like to take their loved one forward. This latter concept, ‘taking the loved one forward’, requires a shift in focus from ‘they are not here anymore’ to ‘how can I take them with me?’. The treatment aims to create intangible continuity: a sense of continuity of the loved one in the patient’s life in an intangible and meaningful way.

The therapist will need to explore with the patient what qualities they felt connected to when they were with their loved one. They will be encouraged to think about their loved one’s best qualities as well as their own qualities that were more alive when they enjoyed time together. Perhaps they felt more light-hearted with their loved one or perhaps more calm or safe. The therapist may ask what the relationship they shared says about their loved one as a person. Importantly, the therapist will guide the patient to discover what their loved one meant to them. Table 7 gives questions to ask to elicit the meaning of the loved one.

**Table 7.** Questions to elicit the meaning of the loved one

What were the qualities you loved about them?
What qualities were alive in you when you were with them?
What does the loving relationship you shared say about them as a person?
What did they mean to you?

*For example, Faye lost her husband to cancer, which had been mis-diagnosed as stress for over a year, meaning that her husband deteriorated rapidly once properly diagnosed. Faye had recurrent images of her husband’s favourite sweater he had been wearing when the doctors told them that the diagnosis was cancer. The worst meaning of the image linked to perceptions of immense suffering coupled with beliefs that the patient couldn’t help him and therefore let him down and was now all alone.*

Guided discovery elicited updating information, including that what Faye loved most was her husband’s warm-heartedness. The worst meaning of the image was updated from ‘*He suffered and was so vulnerable. I let him down and am all alone now*’ to ‘*He isn’t suffering anymore. I am here with our daughter, Tessa. We are okay. I was with him every step of the way. I held his hand, I cared for him, I loved him every minute of our relationship and through his experience with cancer and his last days here. He knew how much I loved him. Now, he is no longer suffering.*’ The next step in imagery transformation would be to bring to mind an image that represents warm-heartedness, the meaning of Faye’s husband.

*4. Discover a new image that represents the updating information.* The fourth step is to guide the patient to discover a new image that represents the updating information. For example, Samira’s new image captured the information that had been discovered with narrative writing of the trauma story. In her new image, she pictured her sister holding their bracelet with two nurses by her bedside. Faye thought that an image of sunshine would best capture the warm-heartedness of her husband.

*5. Transform the image to the new image.* The next step is to call to mind the distressing image, hold it in mind and then to run it on to the new image. It can be helpful in this process for the patient to remind themselves of their new information as they bring the new image to mind.

*For example, Samira called to mind the distressing image of her sister alone in a hospital bed, struggling to breathe. She pictured the image vividly in her mind’s eye. She reminded herself that two nurses were with her when she died and then brought to mind an image of her sister holding their bracelet with the nurses, Stella and Lizanne, overlooking her with kind eyes and gentleness as she lay in the hospital bed peaceful, no longer suffering.*

*Faye brought to mind the distressing image of her husband’s grey sweater. She held it in mind and then pictured sunshine warming the sky and capturing the warm-hearted nature of her husband, no longer suffering.*

At this point, we encourage the therapist to take distress ratings, to ask the patient how distressing the image feels to them now after they have transformed it. A drop in distress is an indication that the updating image is working and distress linked to the loss image is reducing.

*6. Link the image to the relevant moment in the memory* (*memory integration*). The final step is to link the new image to the relevant moment in memory. This is often done together with the patient to update their trauma narrative. It could also be done in an imaginal reliving format. For example, Faye added the updating information and image to her trauma story in a different colour to the rest of her narrative. She wrote:

*My husband, Partha, isn’t suffering anymore. I am here with our daughter, Tessa. We are okay. I was with him every step of the way. I held his hand, I cared for him, I loved him every minute of our relationship and through his experience with cancer and his last days here. He knew how much I loved him. Now, he is no longer suffering and the warmth of sunshine represents his positive, steady continuation of warm-heartedness. Northern Soul captures his upbeat, party, happy sense of self. When I feel the sun on my arm, I am connecting with the warmth of his warm-heartedness touching me, being with me, being all around me. When I listen to Northern Soul, I am connected to his happy, dancing sense of self being with me, all around me, now a part of me.*

Samira called the relevant worst moment in her trauma story to mind in imaginal reliving:

‘*The nurse calls me and says Meira has died. I picture her struggling to breathe and dying alone. I can’t imagine the pain she must have endured, the suffering. Dying alone. I am bereft, I couldn’t be there with her, to hold her, tell her again how much I love her, how grateful I am to have had her as a sister through all those tough times. I feel crushed, lost. I imagine her last moments, I see her struggling to breathe, raspy, tired, wanting to let go, all alone and in pain*…

*… I now know that Meira was holding the bracelet I had given her that we shared over the years when she died. It was wrapped over her fingertips, our loving connection in her hands. I now know that the nurses, Stella and Lizanne, were with her when she died. She did not die alone. She died in the loving kindness of big-hearted nurses with our bracelet that caught our connection over the years. She is no longer suffering. I am picturing in my mind peaceful-looking Meira holding our bracelet with the two nurses, their gentle presence quietening her, making her feel safe, peaceful, no longer suffering*.’

### Trigger discrimination

Then vs Now trigger discrimination is used in CT-PTSD to reduce intrusive memories by discriminating between their triggers in the present context and the similar but different cue in the past trauma. In traumatic bereavement, there tend to be two categories of triggers: triggers that bring back the traumatic memory, which can feel threatening, and triggers of loss memories or happier relationship moments, which elicit intense sadness, loss and grief. Trigger discrimination is used with the former triggers. The therapist and patient will work together to spot triggers that reactivate the patient’s traumatic memories. This includes subtle sensory cues that overlap with those in the trauma (e.g. visual patterns, smells, sounds or body sensations). The therapist will then guide the patient to realise that a memory has been triggered and focus their attention on all the ways the trigger and the current context is different from the memory. The therapist and patient will start by selecting a trigger in session that has reactivated the patient’s trauma memory in their everyday life, first eliciting the similarities and then the differences. The therapist will often find relevant images or audio files or objects that are likely to reactivate a trauma memory. Whilst there may be obvious reminders, such as pictures of a particular hospital or image of a nurse or oncology ward or the smell of disinfectant, there will also likely be more subtle sensory features that trigger memories, such as a certain colour, specific sound, smell, taste, or body position. Sometimes when trauma memories are triggered, patients may not remember the event and only re-experience the same strong emotions (fear, anger, sadness, shame) or body sensations (e.g. nausea or pain) as the past (Wild *et al*., 2020). Therefore, the therapist and patient will work together as joint detectives to spot triggers. Asking the patient to keep a diary of when intrusive memories occur can help to determine the associated triggers.

*Faye described experiencing a distressing intrusive memory of her husband struggling in hospital when she parked her car at the local supermarket. With her therapist, they discovered that for all of her visits to her critically ill husband, the parking lot at the hospital had been lined with wet leaves just like the parking lot at the local supermarket now. The therapist sourced images of wet leaves online and with Faye, they looked at them together in session, first spotting the similarities and then focusing closely on the differences.* Table 8 *shows the Then vs Now table for the trigger of wet leaves.*

**Table 8.** Then vs Now discrimination table for a patient’s trigger of intrusive memories

	THEN	NOW
Similarities	Wet leaves in parking lot	Wet leaves in parking lot
	Dark, autumn evening	Dark, autumn evening
	I am terrified I’m losing Partha	I feel terrified
Differences	I am in the hospital parking lot	I am in the supermarket parking lot
	I am surrounded by illness	There is no illness around me
	People are visiting ill loved ones	People are shopping
	There is an ambulance nearby	There is no ambulance nearby
	Partha is in hospital suffering	Partha is no longer suffering
	Partha is no longer here	I take Partha’s warm-heartedness with me in the sunshine that touches me on a bright day, pictures of sunny skies, images I create in my mind’s eye of rays of sunshine reaching out to me

Following this session, Faye was encouraged to focus on differences between the memory then and the trigger now when she saw wet leaves. In the Then vs Now table, we will often include how the patient is taking their loved one forward with them if one of the core meanings of their trauma memory is that they are all alone now without their loved one.

For triggers (such as personal items, music, places, everyday tasks like cooking) for loss memories or triggers that bring to mind happier memories followed by strong feelings of loss, the therapist will use imagery transformation described in the ‘*Memory updating and imagery transformation*’ section above, encouraging the patient to call to mind the image that captures how they are taking their loved one forward with them in an abstract and meaningful way.

*When Faye saw people horse riding on country roads, she was flooded with memories of her last holiday with Partha where they had gone horse riding. It had been a happy and connecting time and seeing horses had always made her smile until now. Now horses brought back that holiday and filled her with intense sadness. The therapist elicited the meaning of the memory* (‘*The worst thing about the memory is that it reminds me I am all alone now*’) *and encouraged Faye to call to mind her updating image of how she takes Partha forward with her: image of sunshine touching her arm, capturing the meaning of warm-heartedness.*

### Site visit

A visit to the site of the trauma is a core component of CT-PTSD and holds similar purposes for treating PTSD associated with traumatic bereavement as for treating PTSD linked to other trauma. Site visits help the patient to experience that the trauma is over; may lead to discovering new information to update meanings; can help to re-build the trauma memory; and test a belief or concern the patient has, such as that the patient will lose control or be unable to cope. Please see Murray *et al*. (2015) for a detailed guide on how, when, and why to carry out a site visit in CT-PTSD.

Site visits are usually conducted in person with the patient or by visiting the site virtually on Google Street View. They can also be conducted remotely with the patient going to the site alone or with a family member or friend. The therapist can speak to the patient on the phone while they are there to guide them to spot differences between the site at the time of the trauma and how it is now. When using Google Street View, the therapist and patient will walk the route of the trauma virtually, noticing differences between then and now. Google Earth can be used for the parts of the world where Google Street View is unavailable and allows the therapist and patient to view the site from above and zoom in. For traumas that cannot be accessed via Street View, such as inside hospitals or other buildings, the therapist may be able to source images of the outside, and sometimes the interior, as well as access virtual tours of similar buildings. Finally, Google Street View often shows images of the site taken at different dates, which help to show how the site has changed over time and can be used to reinforce the message that the trauma is in the past. Taking photos and reviewing them later is another useful way of consolidating the learning from the site visit. If the patient does visit the site, they can take a ‘selfie’ or, for virtual site visits, screenshots of important images (Wild *et al*., 2020). One complexity to be aware of for events involving multiple deaths is a tendency for some families to create informal memorial items and message boards erected at the site so it is necessary to discuss with patients how they may approach these items in preparation for a site visit.

### Addressing unhelpful maintaining strategies

Patients with PTSD commonly adopt strategies to deal with the sense of threat associated with their trauma, yet which typically keep it going. In traumatic grief, the sense of threat is linked to appraisals that the future is threatening without the deceased. They may ruminate about their loss, trying to make sense of what happened but continue a line of repetitive thinking that leads to no new information. Avoidance is common where patients will suppress traumatic memories or avoid sharing their loss and so never discover that without suppression, memories will come and go or that in sharing their loss, people will mostly extend kindness. Patients may engage in proximity-seeking behaviours to feel closer to their loved one. However, such efforts typically keep their focus on loss. Patients may engage in excessive checking behaviours to feel safer about their surviving loved ones, yet such behaviours keep them focused on danger and paradoxically heighten their sense of threat. Often the first step in addressing maintaining strategies is to elicit their advantages and disadvantages, guide the patient to discover that the disadvantages outweigh the advantages then test the effect of increasing and decreasing the strategies with behavioural experiments.

In working with rumination, the goal is to help clients understand what it is, why it is unhelpful and how they may disengage from this cognitive process. The therapist will guide the patient to discover the effects of ruminating on mood and problem-solving, the types of thoughts that characterise rumination and whether or not such thoughts lead to answers or a productive plan of action. The therapist may use a metaphor of a broken-down car to illustrate the unproductive nature of rumination. The patient is asked to imagine that their car fails before an important journey and that in one situation, they respond with thoughts, such as ‘Why is this happening to me?’ and in another, with thoughts, such as ‘How can I resolve this problem?’. This helps to illustrate how ruminative thoughts affect mood and hinder problem-solving. The therapist will guide the patient to apply this learning to their trauma. It can be helpful for patients to label their rumination when they spot it, e.g. ‘I’m dwelling again’, ‘there goes my spaghetti thinking’). The therapist will then work with the patient to develop a plan to disengage from rumination, putting in place strategies that help them to move onto other activities, such as rebuilding life activities, activities that get them out of their head and into the world when they spot first signs of dwelling.

As rumination is often meaningfully linked to appraisals of self-blame, betrayal and injustice, updating the relevant appraisals can help to reduce rumination. Imaginal reliving or narrative writing, hot spot updating and the use of cognitive therapy techniques, such as surveys, facts from reliable sources, and pie charts, can help to generate updating information. The therapist may make a flashcard with the client that captures the new information, and encourage the client to read their flashcard once when they spot they are dwelling, then try a rebuilding life activity.

*Following the traumatic death of her husband, Faye dwelled about why the doctors had not diagnosed his cancer a year earlier or why she had not pushed him to seek a second opinion when his symptoms were attributed to stress. Through consulting experts and information on throat cancer, Faye discovered that it’s rare in non-smokers and that many doctors would have thought her husband’s symptoms could be stress related. Faye discovered that although the cancer was undetected for a year, the reality is that her husband would not have survived with quality of life even if it had been diagnosed on his first visit to a doctor. A survey she constructed with her therapist helped her to discover that no-one would have questioned the doctor’s impression or arranged a specialist appointment, and no-one thought her trust in the initial diagnosis caused her husband’s death. The therapist created a pie chart looking at the reasons for his death which helped Faye to discover that it was the aggressive cancer that took his life, not her trust in the doctor’s initial diagnosis or deciding against arranging a second opinion. Faye labelled her rumination when she spotted it, then experimented with reading the updating information on her flashcard (see Table 9) once before trying one of her rebuilding life activities, such as looking online for new recipes or doing a 7-minute exercise challenge on her fitness app.*

**Table 9.** Faye’s Flashcard for Rumination

**My Flashcard for Rumination** My ‘Why’ and ‘What if’ thoughts mean my spaghetti thinking has started. I now know that it’s really tough to diagnose throat cancer and it’s rare in non-smokers like Partha. Even if his first doctor’s visit had led to a diagnosis and treatment, he would have had very little quality of life – he would have suffered, he wouldn’t have wanted that, feeding from tubes and in pain. I realise other people would have done what I did and support him with his doctor’s recommendations without pushing for a second opinion. Partha’s cancer killed him, not my care or decision to trust his doctor rather than push for a second opinion. Partha is no longer suffering. I was with him every step of his journey with illness, I loved him, I cared for him, I take him with me in the rays of sunshine that fill my home and touch me on a warm day.

### Relapse prevention

Towards the end of therapy, the therapist will develop a blueprint with the patient, which is a relapse prevention plan structured around six questions. The questions aim to elicit the patient’s learning about how their symptoms developed, what kept them going, what they learned in treatment that helped, their most unhelpful thoughts and the updated alternatives, as well as what they can plan in the future to deal with any setbacks. It will be important for this latter question to think through how the patient will approach anniversaries, paying attention to what CT-PTSD tools may help, such as Then vs Now for triggers, how they may use their updating image, and what rebuilding life activity might then support them. It is important to discuss other potentially strong triggering events such as birthdays. Therapists can explain that often the anticipatory anxiety of these events is worse than the actual day itself. Appropriate planning and preparation for these events replaces anticipatory worry with intentional control of what to do and how to acknowledge the loss as a memory of the death but not a repetition of the death.

## Addressing complexities

Complexities may arise when the patient has had a conflicted relationship with the deceased, when a loved one dies by suicide, the patient is the cause of a loved one’s death, or following pregnancy loss. With conflicted relationships, death by suicide, and when the patient has caused the death of their loved one, complexities arise because levels of guilt are very high. With pregnancy loss, there is complexity because, in relation to imagery and memories, the patient is unlikely to have a clear memory of the deceased from before the loss or to have had a long relationship with them.

### Conflicted relationship

When the patient has had a conflicted relationship with the deceased, it is essential to explore the good times in the relationship as well as the occasions which the patient currently feels distressed about. The therapist may explore what the patient believes overall that each brought to the other’s life to generate a more balanced view of the relationship. The aim of talking about the relationship is to help the patient discover that no matter how their loved one died, the interactions the patient feels guilty about are part of the whole relationship, they do not define the relationship. It is helpful to elicit the meaning of having had difficult times with the deceased: what is the worst thing about having had difficult times? What does the patient fear it means about them as a person? Often the worst meanings link to appraisals about being a bad person or fears that the deceased could not have known how much the patient loved and valued them. Such appraisals can be updated with CT-PTSD tools such as surveys as well as an imagery conversation with the deceased, described below.

### Suicide trauma

When a loved one dies by suicide, guilt is usually the primary cognitive theme. Patients typically believe that they failed to prevent the death of their loved one and that they are therefore responsible. If the patient found their loved one, they will almost always experience distressing images of how they looked when they found them. If they did not find them, they may nevertheless have images of the way they imagined they might have looked that are re-experienced in the same way. In some cases these imagined images are worse than images generated by actual exposure, such as a father who was present at the scene of his son’s death and developed PTSD but the mother, who was not present, experienced intrusive images that were more gruesome and equally distressing.

Treating PTSD linked to suicide trauma will require targeting the cognitive and behavioural processes that are maintaining symptoms and distress with the CT-PTSD treatment components covered in this paper. Before memory updating, the therapist will work with the patient to modify appraisals linked to guilt. The patient would be encouraged to make a list of all the things that they did that were helpful for their loved one throughout their life, not just in their last few months. The therapist will create a responsibility pie chart looking at all factors likely to have contributed to the loved one’s death and consider the patient’s perceived role only after the patient has apportioned a percentage of responsibility to other factors. The therapist will work to transform distressing images following the steps covered in this paper. The therapist may also set up an imagery conversation as there is often distress about things said or not said.

*Nathan died by suicide. Days before he died, he had asked Nathalie, his mum, for money, which she feared he would use to buy drugs, so she had refused and asked him to leave. He had called her names and she had yelled back that he was her biggest disappointment. The next thing she heard was that he had been found hanging from a tree where the family used to camp. Nathalie felt crushed and incredibly guilty. She blamed herself for his death and for not being able to prevent it from happening.*

In this example, the therapist would first loosen then update guilt appraisals and transform distressing images over a few sessions. They may encourage the patient to have a conversation with her son in imagery to say things left unsaid. The patient may also write a letter to their loved one expressing things they had not been able to say while they were alive. If the patient chooses to have a conversation in imagery, then the therapist will guide the patient to picture a place where they used to chat with the deceased. In this instance, the patient said they used to sit in their small garden on occasions on a sunny day and catch up over a beer. The therapist asked the patient to create a clear image of the garden in her mind’s eye and when ready to picture her son sitting opposite her. This is a highly emotional and powerful process, which generally requires a full session. The patient pictured her son opposite her. When she had a good clear picture in her mind’s eye, the therapist asked questions such as ‘When you are ready, what would you like your son to know?’, ‘What else would you like to say to your son?’, ‘How does he respond?’, ‘Is there anything else you’d like to say to your son?’, ‘Anything he would like to say to you?’, ‘Is there anything he needs?’, ‘Is there anything you need?’, ‘How are you feeling?’. When the patient responds with ‘I’m feeling okay or I’m feeling a little better’, then the therapist will ask the patient if there is anything else they need to say or do in the imagery conversation, and then bring their attention back to the present room.

Imagery conversations can offer updating experiences for the patient. Patients know their loved ones well. They have processed and stored in their memories how they respond when they chat. They draw on this when having conversations in imagery. An enormous body of research suggests that work completed in imagery is like having a real experience physiologically (Ehrsson *et al*., 2003; Schnitzler *et al*., 1997). In this way, imagery conversations may be akin to having a real experience and can, in the context of conflicted relationships, offer a warm-hearted corrective experience for the patient.

### Patient is responsible for loss of life

There are occasions where the patient is, to a greater or lesser extent, responsible for their loved one’s death, such as through drink driving, making a decision to turn off life support or an accident caused by the patient. In these cases, it is important to take time to discover highly specific updates relating to how the patient will take forward the meaning of their loved one and this might include restorative measures.

*For example, Kate was celebrating her A-level results with her friends. It was a late night, they went dancing and in the early hours she offered to drive everyone home, she was tired and over the alcohol limit. Kate lost control of the car and her best friend, Tammy, was killed on impact when the car collided with a lamppost. Kate believed that she was 100% responsible for the accident and a pathetic person. She believed there was nothing she could do to take her friend forward with her in a meaningful way.*

In working to update Kate’s worst meanings, the therapist gently probed on the worst outcomes. Kate had driven all of her friends home. The worst outcome would have been if both of her friends had been killed. The therapist helped Kate to see that whilst the death of her friend was due to her driving, they all shared the responsibility of getting into the car together. They also concluded that awareness campaigns on the danger of drink driving may have failed to effectively reach target audiences if Kate and her friends still got into a car whilst over the limit. Kate concluded that there was room to improve such campaigns. Kate made a list of how she had helped Tammy throughout their friendship. With the help of a survey, the therapist was able to guide Kate to discover that although her behaviour on the night of the accident was a contributory factor, it was still an accident and not a sign that she was a pathetic human being. Regarding her friend, Kate said that Tammy was kind, she enjoyed helping other people and looking after their pets. In working on how to take the meaning of her friend forward with her, Kate suggested that she could do some outreach work to improve awareness about how to keep safe from driving when over the limit. She decided to email drink driving courses and charities, sharing her story and offering to speak. Kate updated her story to the following:

*There’s a massive crunch and I pass out, I wake up thinking, I have to get out and check everyone is okay. I see Sonara, she is standing next to the car with her head in her hands. I ask if she is okay and she nods. I look for Tammy. I can’t see her. Then I spot her. She is laying on the ground, her neck looks broken. I rush over to her, I can’t feel a pulse. Omigod, I’ve killed her, she is gone. It’s my fault. I’ve killed her. I am a pathetic person*.

*… I now know that the outcome was worse than I had thought. My best friend died. I now know that it was an accident, I did not intend to kill my friend. It was unlikeable behaviour but it does not make me a pathetic person. I learned from my survey that other people empathise with what happened and do not judge me as a pathetic person. I know now that not everyone was harmed. I know that my friend Sonara was unharmed. I now know although I can’t physically take Tammy with me, I can take her kind nature with me, how she liked to help people. I can contact speed awareness courses. I could share my story so other people, especially young people, know how to keep themselves safe from driving when they’ve been drinking. By doing this work I could save numerous lives over time, many more than were lost in the crash. I can remind myself of this by picturing the heart logo of Brake, the road safety charity*.

We have treated other patients where their violent actions have led to the death of a person who was not a close significant other, such as combatants during conflicts. In these cases we often address appraisals linked to intense shame or guilt and behaviour such as alcohol or drug abuse to suppress negative beliefs about the self and past destructive actions. As therapists we do not collude with strategies that minimise the seriousness or the effects of the patient’s actions causing death, but we help patients to consider new strategies such as how to make restitution and a make a positive contribution to society in the future. We will cover these more specific issues in a separate paper.

### Pregnancy loss

PTSD related to pregnancy loss of a full-term baby is treated in a similar way as PTSD related to loved adults who have died. The exception is early miscarriages and terminations where the parents will not have met their baby, or the growing foetus may not yet have taken the shape of a baby. When updating memories and images, the therapist will first need to discover what the patient hoped or felt their baby would be like, how they saw their baby in the early stages, and importantly what the baby brought to their lives, what they represented and what they will miss. This information helps to inform a potential image that could capture the meaning of their baby they can take forward. For step-by-step guidance on delivering CT-PTSD for birth trauma, please see Kerr *et al*. (2022).

## Discussion

With traumatic loss, the patient commonly reports that their most dreaded and frightening belief has come true and their loved one has died, often in horrendous circumstances. PTSD is common after such trauma and interrupts the natural course of grieving, meaning that PGD is also common in these circumstances. CT-PTSD can and is successfully applied to PTSD arising from loss trauma. In this paper we have described the core components of CT-PTSD and how to apply them to traumatic bereavement. Notable differences to treating PTSD arising from bereavement trauma compared with trauma where there is no loss of life is that CT-PTSD will almost always incorporate imagery transformation as part of memory updating. Rebuilding life activities are planned rather than reclaiming life activities. Finally, addressing cognitive themes almost always precedes memory updating. When traumatic loss involves loss of a conflicted relationship, suicide trauma, or circumstances where the patient has caused the death of their loved one, conversations in imagery may take place to elicit a corrective experience in which the patient can say things that have been left unsaid and connect to their internalised memory of their loved one, accessing their knowledge base of how they would respond.

CT-PTSD suggests that the patient may carry their loved one forwards in intangible ways and this appears to reduce the intensity of loss and sadness. Yearning associated with bereavement trauma appears reduced because the patient has a sense they are moving forward with their loved one, albeit in an abstract way, and may be less driven to search for them as a result. Whilst this is our sense of how intangible continuity helps to reduce the intensity of feelings, future research is needed to test such hypotheses. One avenue would be to determine if increases in the sense of continuity result in reductions in the strength of loss, sadness and yearning. CT-PTSD differs from existing cognitive behavioural approaches to traumatic grief reactions (e.g. Boelen *et al*., 2006) in several ways (see Ehlers, 2006). Importantly, working with meanings of the loss may include creating a sense of continuity with the loved one rather than focusing on the irreversibility of the loss. Emerging research is attempting to determine whether or not continuing bonds with the deceased facilitates adjustment after loss. A review (Stroebe and Schut, 2005) directed at answering this question suggests that continuing bonds may be correlates of healthy grieving. Research is required to determine whether such bonds lead to healthy adjustment. Such research will need to define the nature of continuing bonds. Many studies refer to continued bonds as, for example, keeping a belonging of the deceased, reminiscing about the past or talking to the deceased, which differ to the CT-PTSD conceptualisation of carrying the meaning of the deceased forwards.

Transforming traumatic images associated with loss appears to reduce their distress and, delivered in the context of CT-PTSD, is associated with symptom reduction. Since imagery transformation aims to update the worst meanings of the images and linked memories, it appears to make them less threatening, leading to two outcomes. The first is that the patient will be less likely to respond to intrusive loss memories with suppression or dwelling, which may facilitate emotional processing and integration of the loss memory with the patient’s broader autobiographical memory base. Second, updated appraisals associated with the image and memory may lead to reductions in PTSD symptoms, facilitating recovery from the disorder and a more balanced outcome with grief. Research has indeed demonstrated that changes in appraisals with CT-PTSD precede symptom change (Kleim *et al*., 2013).

As PTSD appears to hinder the grieving process, recovering from PTSD should support the natural process of grieving. CT-PTSD for traumatic bereavement facilitates a sense of continuity with the loved one rather than reinforcing the sense of finality associated with their death. The therapy introduces *intangible continuity*, the idea that the patient can take their loved one forwards in what they do and how they think because of the intangible ways they were influenced by them. In this way, the patient carries their meaning forward. Thus, the treatment may offer a sense of comfort as there is no requirement to stop thinking about the person who died but rather, to think about the loss differently, focusing on how to connect and honour the important ways in which they were influenced by their loved one, which may give a sense of moving forward over letting go. People struggling with their grief frequently say that what they fear most about letting go of grief is the thought of letting go of their loved one. Perhaps shifting focus to how the patient can carry the meaning of their loved one forward supports the patient in moving forward with their grief.

CT-PTSD has been delivered to patients experiencing a range of trauma, including traumatic loss, and has been evaluated in several RCTs (Ehlers *et al*., 2003; Ehlers *et al*., 2005; Ehlers *et al*., 2014; Ehlers *et al*., 2020) as well as in routine clinical practice (Duffy, Gillespie and Clark, 2007; Ehlers *et al*., 2013; Ehlers *et al*., 2023) and is associated with high rates of recovery. However, to determine the efficacy associated with the different components of CT-PTSD as delivered for traumatic bereavement, a dismantling study is required or a study of an experimental design in which the procedures are evaluated in isolation. Qualitative feedback from patients who have had CT-PTSD for traumatic bereavement suggests it is a powerful process that, when the worst has happened resulting in unimaginable suffering, leads to a lasting and, as the patient quoted at the beginning of this paper says, ‘…a really good result’.

### Conclusion

Most people will face loss in their lives, whether it is loss of a loved one through trauma, illness, geographical distance or the end of a relationship. CT-PTSD suggests the way forward is to move forward with loss, to spot the meaning of what has been lost and to bring that meaning back into patients’ lives whilst recognising what has not been lost. In doing this, the therapy creates a new relationship to loss. It is no longer about letting go or saying good-bye. It is about acknowledging that the patient can take their loved one forward in an abstract and meaningful way. In this way, CT-PTSD creates a sense of continuity today with what has been lost in the past. By conceptualising loss like this, the treatment frees patients from the fear and despair associated with loss trauma, rooting them in what matters most – the meaning they give their lives and the relationships in them.

## Data Availability

Data availability is not applicable to this article as no new data were created or analysed in this study.

## Key practice points

1. CT-PTSD can be successfully applied to PTSD arising from traumatic bereavement.
2. CT-PTSD introduces *intangible continuity*, the idea that patients can take their loved one forwards with them in an abstract and meaningful way.
3. CT-PTSD for bereavement trauma is tailored to the life-changing loss in a number of ways: (1) space is given at the start for talking about the person who died and the client’s relationship with them; (2) memory updating typically incorporates imagery transformation; (3) rebuilding life activities are planned; and (4) cognitive themes are almost always addressed before memory updating.
4. When traumatic loss involves loss of a conflicted relationship, conversations in imagery may take place to elicit a corrective experience with the loved one.
5. Transforming loss images involves six steps, which include eliciting and updating worst meanings then capturing the updated meaning in an image before incorporating it into the trauma memory. The updating image can also be called to mind in response to intrusive memories.
